# Methodological issues in epidemiological studies of periodontitis - how can it be improved?

**DOI:** 10.1186/1472-6831-10-8

**Published:** 2010-04-21

**Authors:** Roos Leroy, Kenneth A Eaton, Amir Savage

**Affiliations:** 1 Catholic University Leuven, Leuven, Belgium; 2 University College London Eastman Dental Institute, King's College London Dental Institute and University of Kent, Canterbury, UK; 3 Defence Dental Services, Dental Training School (DTS), UK

## Abstract

**Background:**

This position paper was commissioned by the European Association of Dental Public Health, which has established six working groups to investigate the current status of six topics related to oral public health. One of these areas is epidemiology of periodontal diseases.

**Methods:**

Two theses "A systematic review of definitions of periodontitis and the methods that have been used to identify periodontitis" [1] and "Factors affecting community oral health care needs and provision" [2] formed the starting point for this position paper. Additional relevant and more recent publications were retrieved through a MEDLINE search.

**Results:**

The literature reveals a distinct lack of consensus and uniformity in the definition of periodontitis within epidemiological studies. There are also numerous differences in the methods used. The consequence is that data from studies using differing case definitions and differing survey methods are not easily interpretable or comparable. The limitations of the widely used Community Periodontal Index of Treatment Need (CPITN) and its more recent derivatives are widely recognized. Against this background, this position paper reviews the current evidence base, outlines existing problems and suggests how epidemiology of periodontal diseases may be improved.

**Conclusions:**

The remit of this working group was to review and discuss the existing evidence base of epidemiology of periodontal diseases and to identify future areas of work to further enhance it.

## Current Problems

The methodology for periodontal studies remains elusive [3]. A fundamental prerequisite for any epidemiological study is an accurate definition of the disease under investigation. Unfortunately in periodontal research, uniform criteria have not been established [4]. Because of methodological problems the data used to assess treatment needs for periodontal diseases have been of questionable value and are not comparable [2]. Comparison of effect of risk factors (Odds Ratio, Relative Risk) between studies is hard [5]. A systematic review of the literature discovered that only 15 studies, out of 3472, gave a definition of periodontitis and indicated how it was measured. The criteria for a diagnosis of periodontitis ranged from 3 mm - 6 mm probing pocket depth and for clinical attachment loss (as an indicator of periodontitis) from 2 mm - 6 mm [6]. The reviewed studies used measurements at different sites using different measurement tools [6].

## Current Situation

In general, periodontists tend to treat individual patients (with an array of clinical techniques, including placing implants) and are rarely involved in the epidemiology of periodontal diseases of the general population. Dental public health workers often come from a background of paediatric dentistry and may place more emphasis on the epidemiology of dental caries experience. These factors and others have conspired to inhibit improvements in epidemiology of periodontal diseases.

### Epidemiological data on periodontal health/disease

Accurate epidemiological data are a prerequisite to:

• identify people at risk in the population,

• assess the efficacy of preventive strategies and curative therapies at a population level,

• carry out work force planning.

Moreover, they are necessary in order to:

• evaluate the interplay with risk factors of periodontitis,

• assess the interaction between periodontal health/disease and systemic diseases,

• assess the impact of periodontal diseases on quality of life.

Two theses "A systematic review of definitions of periodontitis and the methods that have been used to identify periodontitis" [1] and "Factors affecting community oral health care needs and provision" [2] formed the starting point for this position paper.

### Case definition of periodontitis in epidemiological studies

A recent review of analytical epidemiology revealed a conspicuous lack of uniformity in the definition of periodontitis in epidemiological studies [5]. This problem has also recently been highlighted for periodontal research by Preshaw [7], who suggested open discussion to firmly establish criteria for defining a periodontitis case in research. Researchers have historically used an array of clinical signs and symptoms such as gingivitis, bleeding on probing, pocket depth, clinical attachment loss, radiographically assessed alveolar bone loss and even tooth loss, the ultimate endpoint of periodontal disease [8-11]. Further complications are posed by the fact that in some studies multiple disease indicators such as pocket probing depth (PD) and clinical attachment level (CAL), both representing current pathology and cumulative tissue destruction respectively are used [12]. The situation is further confused by the variation in threshold values used in defining cases regardless of the criteria used.

In 2003 the Centres for Disease Control and Prevention and the American Association of Periodontology appointed a working group to investigate and develop methods for periodontal disease surveillance at population level using self-reported measures. As part of this task, the group recognized that in order to compare accuracy of self-reported measures across studies, a robust gold-standard definition (based on clinical exam) including a consistent definition of periodontitis was required. This classification defines severe and moderate periodontitis in terms of PD and CAL to enhance case definitions and it also demonstrates the importance of thresholds of PD and CAL and the number of affected sites when defining periodontitis.

The case definitions that were proposed are tabulated below [13].

< Table 1. Case definitions for periodontitis - about here>

**Table 1:** Case definitions for periodontitis (Page and Eke, 2007)

Disease Category*	CAL				PD
Severe Periodontitis	≥ 2 interproximal sites with CAL ≥ 4 mm (not on same tooth)		and		≥ 1 interproximal site with PD ≥ 5 mm
Moderate Periodontitis	≥ 2 interproximal sites with CAL ≥ 4 mm (not on same tooth)		or		≥ 2 interproximal sites with PD ≥ 5 mm (not on same tooth)
No or mild Periodontitis	Neither "moderate" nor "severe"				

* Third molars excluded

Page and Eke (2007) further state that it is hoped that these definitions will serve as standard for population-based surveillance of moderate and severe periodontal disease for the future, which will bring some uniformity to case definitions of the disease across studies [13].

In Europe the following two threshold level criteria for the diagnosis of periodontitis were proposed during the 5^th ^European workshop in Periodontology [14]:

1. The presence of proximal attachment loss of ≥ 3 mm in ≥ 2 non-adjacent teeth

2. The presence of proximal attachment loss of ≥ 5 mm in ≥ 30% of teeth

The first threshold level enabled the application of a sensitive case definition (including incipient cases) and the second allowed a more specific case definition in order to identify only cases with substantial extent and severity [14]. The 3 mm threshold is based upon studies on incremental attachment loss measurement, where the error of the recording method was calculated at 2.5 mm. The report emphasized that attachment loss should be the primary outcome variable used in studies of risk factors for periodontitis. However, it stressed that periodontitis cannot be reflected by measurements of only a single variable such as attachment loss or bone loss but required the additional measurements of bleeding on probing and/or pocket depth.

The authors further emphasize that the proposed criteria are not designed for the assessment of prevalence of periodontitis across nations and/or age groups, the focus is rather to identify risk factors. Hence, the question arises which of the case definitions described above can be used in periodontal epidemiological studies.

### Clinical versus radiographic examination

In most epidemiological studies oral examinations are not undertaken at the same time as routine dental examinations or treatment sessions. As a result radiographs are rarely available. Furthermore exposing subjects to radiographic examination solely for epidemiological purposes is considered unethical in most countries. Consequently it is generally not possible to assess radiographically attachment loss/alveolar breakdown due to periodontal disease. In addition, radiographs only provide a two-dimensional image of a three-dimensional situation.

In field studies the threshold of bone loss measured from the alveolar bone crest to the cementum enamel junction (CEJ) has ranged from 1 mm to 3 mm [6]. A clear case definition for this radiographic parameter has not been defined.

Another issue that has to be taken into consideration is that the periodontal attachment may physiologically move apically in adult subjects with occlusal attrition (continuous eruption or loss of antagonistic contact). Therefore suggestions have been made that periodontal attachment studies should be age related [15].

### Full versus partial mouth recordings

The choice of full versus partial mouth recordings (e.g. half mouth or index teeth) of the clinical data obtained needs to be addressed. Full mouth assessments (as performed in a number of epidemiological studies including [16-19]) provide the optimal examination of periodontal conditions [6]. Although it is desirable to record as many sites as possible to increase the probability of detecting disease prevalence, one of the main drawbacks of full mouth assessments is that it can be time consuming.

Partial mouth assessments have the marked advantage of being quick. Important procedural aspects in population studies are short examination times and the requirement to minimize subject discomfort. Partial mouth assessments maximize the number of people examined in the time available and encourage subjects to comply with a study protocol [17].

Nevertheless, they do have the potential to underestimate the prevalence of periodontal breakdown in populations with less susceptibility [10] or overestimate the prevalence when the teeth selected are first molars and lower incisors [20]. This is in agreement with Eaton et al. (2001), who concluded that the use of index teeth in epidemiological studies which include young adults may result in an underestimation of the prevalence of early periodontitis and an overestimate of the extent, based on the measurement of lifetime cumulative attachment loss [21]. In addition, Susin et al. (2005) suggested that the bias in the assessment of attachment loss should be considered when selecting partial mouth recordings in large surveys [22]. They also suggested that a correction factor, designed to adjust for the partial assessment bias, should be calculated and reported so that comparisons of results with other surveys could be made more meaningful.

Therefore it can be argued that to account for the limitations in partial mouth assessments, a correction factor should be calculated by performing full mouth assessments on a certain percentage of subjects and comparing the results with those obtained from partial mouth assessments [1].

In addition to the above, there is also the problem of 'clustering of data' as several scores are collected in one subject, which has important analytical consequences. Within an individual, teeth are not independent from each other. While currently available tests (e.g. Wald test) can be applied for testing inter-subject differences (e.g. differences between women and men), standard statistical software cannot be used when intra-subject comparisons (e.g. comparisons between contralateral and antagonistic teeth) are envisaged. Statistical models applicable for dependent data are then needed. If the dependence of clustered observations is overlooked, point estimates may be similar, but variance estimates may be drastically different. Hence, the confidence intervals for the naïve model are narrower, resulting in an increased risk for type I error and erroneous rejection of the null hypothesis [23]. Also, the multilevel nature of data (several sites per tooth and several teeth in one mouth) should not be overlooked.

### Indices

Ideally, an index should be simple to understand and easy to learn how to use [24]. Savage echoed the advice of previous workers in the field and enumerated the other prerequisites for good and efficient indices [1]. Indices should be objective and not be susceptible to the examiner's opinion. They should have clear cut categories that make it easy to make a decision as to which category a condition should fit into. An index should be valid possessing, in statistical terms, good sensitivity and specificity. Furthermore, the ideal index should also be reliable and reproducible with no variations as a result of internal flaws within the index and give the same result if the condition being assessed has not changed. It should also be able to detect small changes and should be able to measure changes in either direction, that is, whether the condition being measured improves or deteriorates. Finally, it should be acceptable and free of discomfort for patients/subjects, with the length of time to complete any assessment and examination taken into consideration.

It is important to note that most indices give historical information on previous disease rather than actual presence of disease. Moreover, few periodontal indices measure individual clinical features or variables. Instead they classify or grade them according to various criteria [2]. This frequently causes difficulties in two areas. The first is the application of the index concerned in a consistent manner, not only in epidemiological surveys but also in clinical practice and research. The second is the inappropriate consolidation of index scores to produce mean scores for variables such as plaque and gingival inflammation, since such scores are not arithmetic and do not represent actual indices [2].

### Community Periodontal Index of Treatment Needs - CPITN

#### The Development and Uses of CPITN

CPITN was proposed by WHO in 1977 as an index to evaluate the periodontal treatment needs of populations [25]. It was subsequently included in WHO O*ral Health Surveys - Basic Methods*, 3rd Edition (1987) and 4^th ^Edition (1997) [26]. Relatively recently, the CPITN has been renamed as the Community Periodontal Index (CPI) to denote its use as an epidemiological tool rather that as an aid to treatment planning. However, historically a number of studies have suggested that it can be used as an indicator of both the prevalence of chronic inflammatory periodontal disease (CPID) and periodontal treatment needs [27-29]. The main advantage of the index is that it is easy to use [30]. As a result of this feature and the promotion of the use of the index by the WHO, it has been widely used internationally for several years. Nevertheless, the index does have a number of limitations, which call into question its use in the future.

#### Limitations of CPITN

The index is based on a hierarchical concept of the progression of periodontitis which implies that a tooth with a score of 3 or 4 (a pocket present) should also have calculus present (score 2) and bleeding (score 1). The validity of this assumption has been challenged [30,31]. In a Norwegian population, 30% of teeth with calculus did not present with bleeding and 25% with deep pockets (score 4) and bleeding did not have any calculus present [32]. In a Japanese population, bleeding was absent in 47.5% of sextants with a CPITN score of 2 for calculus [33]. In Hong Kong, Holmgren and Corbet (1990) reported a similar finding and concluded that the presence of calculus without bleeding at a sizeable proportion of index teeth without pockets, questioned the assumption of the CPITN that there is a close concordance between calculus and periodontal inflammation [34]. Further limitations of CPITN are that it does not measure tooth mobility or attachment loss [29,35] or furcation involvement. Gera (2000) has suggested that in populations with access to periodontal care, teeth may experience considerable gingival recession following therapy and have minimal pocket depth leading to CPITN scores of 0 or 1, when there has actually been considerable past attachment loss, thus leading to underestimates of the extent and severity of periodontal destruction that has previously occurred in their mouths [36]. There are also doubts about the ability of any technique that examines the periodontium around just a few teeth to reflect the true state of periodontal health or disease in the mouth concerned. These limitations have led to the value of the CPITN being questioned as a reliable epidemiological tool [37,38].

A number of modified indices, based on the CPITN, have therefore been developed in an effort to address the limitations. However, they also all have some limitations. The limitations relating to attachment loss leading to gingival recession and furcation involvement were addressed by the development of the Basic Periodontal Examination (BPE) index by the British Society for Periodontology [25,39]. These two variables are recorded by an asterisk in the score for the sextant concerned. A further refinement has been included in the Periodontal Screening and Recording Index [40,41]. This index also adds an asterisk to sextant scores not only when furcation involvement and gingival recession (> 3.5 mm) are present but also if there is tooth mobility. In the United Kingdom, the Defence Dental Agency has used a modification of the CPITN, the Periodontal Index for Treatment (PIT) in which there is no score for calculus [42].

### Clinical Attachment Loss

Clinical Attachment Loss (CAL) and/or Probing Pocket Depth (PD) may well be the best indicators to use in epidemiology of periodontal diseases. CAL gives an indication of past periodontal disease and PD may give better indication of current disease status [43,44]. Consideration of varieties of CAL such as lifelong clinical attachment loss (LCAL) should be considered for younger age groups, who may have a different threshold of disease [21].

Consideration should also be given to other clinical parameters such as bleeding on probing.

### Errors intrinsic to periodontal probing

Probing pocket depth and attachment loss, often the major outcome variables in periodontal (epidemiological and clinical) studies, are both measured with a periodontal probe. A number of complicating factors associated with periodontal probing frequently render these two measurements unreliable. They can be summarized as follows:

• The extent to which a probe penetrates into a given pocket. This can vary because inflammation at the base of a pocket reduces resistance to a probe tip and may permit it to penetrate the base of the pocket [45]

• The diameter of the probe tip [46]

• The tine (part of the probe with markings) [47]

• The probing force used is a further major factor influencing probe penetration [48,49]

• The angulation of the probe tine to the pocket wall [50]

• The accuracy or otherwise of markings on the tine, which even in the same batch from a production line, can vary by more than 0.5 mm [51]

• Experience of the examiner

• Presence of (overhanging) restorations

It has also been observed that probe tines, both parallel and tapering, with the same tip shape and diameter (0.5 mm) differed by 1 mm in probing depth assessment of minimally inflamed periodontal pockets [47]. It has been suggested that the use of automated or electronic probes might improve consistency. Pihlstrom (1992) has classified periodontal probes into three generations [52].

• *First generation*: non-pressure controlled (manual) with visual data recording,

• *Second generation*: pressure controlled with visual recording,

• *Third generation*: pressure controlled with direct computer data capture.

Breen et al. (1997) reported mean probing differences ≥ 0.5 mm between pocket depth and attachment levels when these variables were recorded at the same sites with first and second generation probes and a lesser difference when the means of the measurements obtained with first and third generation probes were compared [53]. The highest prevalence of ≥ 4 mm and ≥ 6 mm pockets occurred when the first generation (manual) probe was used. It should be noted that the examinations were performed by only one examiner in only six patients with chronic adult periodontitis.

In a subsequent study this group reviewed 23 studies of site-specific attachment level changes and found that all three generations of probes had been used within the 23 studies. A wide variability of probe types had been used. There had also been a wide variety of recording protocols and methodologies employed for data analysis [53]. Only two of the studies reviewed [54,55] included more than 100 subjects and none were epidemiological surveys. Breen et al. (1999) concluded that valid comparisons between studies were therefore rarely possible [53]. It should be remembered that, to date, large-scale epidemiological surveys have invariably used first generation probes. Given the tine faults as previously discussed, definitive inferences from the data must be considered of questionable value.

In terms of reproducibility of measurements for probing pocket depths and attachment loss, studies performed by Perry et al. (1994) and Tupta-Veselicky et al. (1994) suggested that there were no significant advantages in the use of second or third generation probes [56,57]. This was also confirmed in a systematic review of clinical trials evaluating the reproducibility of manual (MP) and electronic probes (EP) in the measurement of clinical periodontal attachment level (AL) in untreated periodontitis subjects [58]. These authors concluded that manual (MP) and electronic probes (EP) showed a tendency to have similar reliability in the measurement of CAL in untreated periodontitis subjects when used by a calibrated examiner, but note that this finding is not supported by strong evidence. On the other hand, Magnusson et al. (1988) and Osborn et al. (1990) found greater reproducibility when no first generation probes were used [59,60].

However, the practicality of using second and third generation probes in field studies can be questionable.

### Inter- and intra- examiner consistency

In epidemiological studies clinical measurements are invariably collected by more than one examiner, raising the issue of inter-examiner variability in measurements.

The World Health Organisation recommendation in "O*ral Health Surveys; Basic Methods"(4^th ^Edition, 1997) *is that examiners taking part in epidemiological surveys should attend training and calibration sessions that should last for at least four to five days and should lead to intra- and inter-examiner agreement over scores in the range of 85 - 95%. In a limited review of the literature it was observed that only a limited number of studies report a measure of agreement for pocket probing depth and attachment loss measurements [2].

If a measure of agreement between examiners is reported, a high value of Cohen's Kappa coefficient indicates strong agreement between examiners' scoring [61]. It is generally advisable to set limits for Kappa "a priori" rather then apply them ruthlessly [62].

It is also important to recognise the limitations of this statistic [63]. A kappa score will not inform whether any disagreement is caused by just one examiner consistently scoring high or low, moreover the accuracy of kappa is influenced by the disease prevalence. When a gold standard or benchmark score is available, it is recommended that sensitivity and specificity calculations of each examiner are undertaken and measured against this gold standard (or benchmark score) [63].

## Conclusions

It is clear that there is a strong need for future uniformity in the design of epidemiological studies on periodontal disease. Such studies need to be designed to allow the resulting data to be readily compared with those obtained from other studies. Furthermore, as better understanding and new data emerge, the design of periodontal epidemiological studies will need updating to reflect these advances.

## Recommendations for the future

1. It is suggested that the first step in this process of achieving homogeneity in periodontal research should be the use of a uniformly agreed measure and a measuring tool that clearly defines the disease threshold and the surveyed area. This may be difficult to achieve due to the wide variation of probes available and individual operator preference.

2. If partial mouth assessments are made then a correction factor should be calculated to account for possible differences between partial mouth and full mouth assessments.

3. There should be a requirement for rigorous training and calibration of the examiners and the nature of this training and calibration must be fully reported.

4. Finally, in order to truly reflect disease activity, and reiterating previous literature on the subject, the combined use of CAL, PD and bleeding on probing should be considered as the three key variables to be assessed in future epidemiological studies on periodontal disease.

5. In order to achieve these goals it will be necessary for all those involved in periodontology and dental public health to work together at a global level.

## Competing interests

The authors declare that they have no competing interests.

## Authors' contributions

RL did additional literature search, drafted the manuscript and co-chaired the working groups in which the position paper was discussed (Heidelberg, September 2008; Stockholm, June 2009 and Tromso, September 2009). KE performed literature search (within the frame of his PhD thesis), co-chaired the working groups in which the position paper was discussed and critically read the manuscript. AS performed the initial literature search (within the frame of his thesis) and critically read the manuscript. All authors read and approved the final manuscript.

## Pre-publication history

The pre-publication history for this paper can be accessed here:

http://www.biomedcentral.com/1472-6831/10/8/prepub
